# Books and Authors

**Published:** 1912-01

**Authors:** 


### BOOKS AND AUTHORS

**Text Book of Physiology, Wm. H. Howell,** Ph.D., M.D., Baltimore; published by W. B. Saunders Co., Philadelphia and London, 1911. 1018 pages, illustrated, cloth, $4.00, half morocco, $5.50.

This book, large as it is, is net physiology. The usual lengthy discussions of histology, embryology and chemistry are conspicuous by their absence, though necessarily alluded to under various subdivisions. The author also departs from the ordinary in starting with the phenomena of muscular contractions. Some recent statements as to the electric nature of nerve-impulse, are, apparently refuted. The older, conservative statements that nerve impulse cannot be electricity because of its slowness, are corroborated and the interesting facts that nerve impulses travel at different rates in different types of animal are developed. What nerve force is, remains uncertain. The brief discussion of diet and calories is lucid, though we are somewhat disappointed not to have found a general law for deriving the caloric value of a proximate food principle from its chemic formula. Neither do we find a clear and elaborate tabular presentation of the nine or ten special senses. The exact nutritive value of nutritive enemata is not given, though absorption and digestion in the large intestine are discussed. The experiments cited with regard to the effect of maintaining the congenital condition of alimentary sterility, are contradictory and the writer is left in doubt as to whether intestinal bacteria are necessary, harmful or merely inevitable. A great many similar disappointments are in store for the reader. To put it otherwise, the author does not let his imagination run riot; he does not distort the truth nor anticipate discovery in order to make a subject complete and logical. He has written not scientific fiction but cold fact. The size of the work in the absence of speculation, lines written for the gallery, and the minutiae of vivisectors indicates its genuine thoroughness.

---

**Diseases of Infants and Children,** by **Henry Dwight Chapin,** A.M., M.D., and **Godfrey Roger Pisek,** M.D., New York. Published by Wm. Wood & Co. 1911. 636 pages, 181 illustrations, 11 colored plates. Cloth, $4.50.

The most striking feature of this work is the large amount of interesting material not to be found in other standard text books on the subject and which the average physician does not know. For instance, a few weeks ago, a friend informed us of a case of abortion of a foetus without an umbilic cord. This seemed to both of us, at first thought, impossible and, at least, unprecedented. Chapin and Pisek's book opened by accident to an illustration of a mammary (kangaroo) foetus and a full explanation of marsupialism, the foetus being without placental connection, and attaching itself after birth to the teat in such a way as to afford a substitute for placental nutrition. The illustration of the six phases of nutrition, from the ovum drawing on its yolk to the adult seated at a well spread table—the fourth phase of breast feeding being included—is almost of the nature of a cartoon. In fact, the usual consideration of paediatrics is enlivened in a most original and practical manner.

---

**Treatment of Fractures,** 7th edition, **Dr. Charles L. Scudder,** Boston, published by W. B. Saunders Co., Philadelphia and London, 1911. 708 octavo pages, 990 original illustrations, $6.00 in buckram, $7.50 in half morocco.

For good measure, the author adds what he very modestly calls "Notes upon a few common dislocations." A book that furnishes 14 illustrations to every 10 pages, and original illustrations at that, evidently comes close to being an object lesson. It would be a valuable clinical atlas, with no text at all. With a very lucid and full, though concise text, it lacks only the master's voice to make it a systematic series of clinics. The bones themselves, normal and variously fractured, are shown; when necessary, the relationship of muscles, viscera, vessels, etc., is also shown and almost every conceivable lesion of viscus, nervous system, and other structures, including the results of contusion and of infections liable to complicate fractures, is also depicted. *X*-ray appearances are systematically given and dressings and methods of manipulation are both described and illustrated. The book is so nearly perfect that we are tempted to express the fear that an incompetent man, with no experience, might see a fracture case, surreptitiously consult this book and, in five minutes, spent in reading and glancing at the pictures, feel prepared to deal as an expert with the most difficult complicated fracture. Some day, the author may regret that he has left nothing for an eighth edition—but the man who can bring out such a work, is always a harsh critic toward himself and can doubtless find something to improve and more material to add. Rarely does one encounter a book that appears to be so nearly the last word that can be written on a subject.

---

**A Quaint Tale of the Origin of Syphilis.**

When you have finished Fracastor's tale—THE ORIGIN OF SYPHILIS—you will lay it down and say: "Now that's a book worth while." Little did Fracastor dream way back in 1530 when he was writing his famous work, that in 1911 an American publisher would issue an English translation.

Finishing his delightful mythologic origin of syphilis, Fracastor plunges into a description of its symptoms and manifestations, which, for word painting cannot be surpassed. Possessed of a keenly observant mind, and the wielder of a pen that records his thoughts with cameo-like distinctness—Fracastor has given us what is truly a masterpiece. While science has lately rewritten the story of syphilis, and may rewrite it again a generation hence, yet Fracastor's book will live for, verily, it is one of man's imperishable works. The book may be secured from the Philmar Company, Fidelity Building, St. Louis, Mo., for two dollars.

---

**Veterinary Bacteriology,** by **Robert E. Buchanan,** Ph.D., Professor of Bacteriology, Iowa State College of Agriculture and Mechanic Arts, W. B. Saunders Co., Philadelphia, 1911. Octavo, 516 pages, 214 illustrations, cloth, $3.00.

As is customary, the term bacteriology includes the discussion of yeasts, molds and protozoa, as well as bacteria, and the infections affecting the domestic animals generally, fowls, etc., are treated fully. So far as the general scientific basis of bacteriology is concerned, and the description of technic, this work is equally valuable to the student of human pathology. Much practical information is given as to the preparation of antitoxins, vaccines, etc. We wish that every physician could read this work so as to obtain a definite idea of the infections of human beings likely to be communicated to the lower animals and vice versa.

This is a matter regarding which many erroneous ideas prevail, among them the notion that the lower animals are, in general, highly resistant to disease, as compared with man.

---

**Diseases of the Stomach,** by **Max Einhorn,** M.D., New York, published by Wm. Wood & Co., New York, fifth edition, 1911. 531 pages, 112 illustrations, cloth, $3.50.

While smaller than many of the standard stomach books, Einhorn's is especially interesting and valuable on account of the many inventions of his own fertile and ingenious brain. For example, we may mention his vacuum bottle for peptic tests with ricin; his radium holders, pyloric dilator, gastroscope, microscopic study of gastric exfoliations, etc. Such studies not only afford definite means of investigation but stimulate the reader's powers of observation and scientific imagination. There is only the necessary minimum of established and trite material in Einhorn's book, and a maximum of the novel and interesting. Best of all, the author's clear, forcible though never dogmatic style, invites criticism. For instance it seems to us that hyperchlorhydria should be definitely limited to an excess, of notable degree, of hydrochloric acid itself and we should be loath to include under this term all cases with a total acidity of over 60 degrees unless the HCI itself were disproportionately increased. So, too, achylia gastrica deserves a fuller treatment, from the standpoint of a wider and less personal experience and we would like to see a positive statement regarding Knapp's contention that it is the same as or inseparably connected with pyloric relaxation. The book would be improved by greater attention to nomenclature. These, however, are very minor defects. It is a strong, virile treatise, which makes one think, and argue with himself and, in most instances, the more one thinks and argues, the more likely he is to come around to the position of the author.

---

**Collected Papers by the Staff of St. Mary's Hospital, Mayo Clinic,** 1910. Published by W. B. Saunders Co., Philadelphia and London, 1911, 633 pages, 291 illustrations, cloth, $5.50.

The papers consist both of elaborate reports of cases observed, with thorough pathologic and clinical study and statistic grouping of material but of essays on miscellaneous subjects, as notes of Italian Surgery, the Commencement address by Dr. W. J. Mayo at Rush Medical College and a number of monographs on matters not entirely of a surgical nature. We need scarcely say that the work is characterized by thoroughness and large practical experience.

**Refraction and Visual Acuity** by **Kenneth Scott,** M.D., C.M., F.R, C.S. Edin———Rebman Company, New York, Publishers.

This treatise is written primarily for the general practitioners of Great Britain and the British possessions, therefore much that will be of value to them is not very useful to the American physi-cian. For instance, the visual requirements of the Dublin and South Eastern Railway, the Central South African Railways, or the Great Indian Peninsular Railway, though of importance to the officials conducting such examinations, are hardly likely to prove of much interest to the general practitioners of the United States.

The writer is a firm believer in retinoscopy, and in a simple and clear manner points out its many advantages. The reviewer having constantly used this method for twenty years is in substantial agreement with his views. However, on one point—the use of the plane mirror—exception must be taken to the statement that "no after calculation is required; as it is the strongest convex lens that still preserves the hypermetropic movement before it is reversed that indicates the amount of error there, and in myopia it is the weakest concave glasses first producing the hypermetropic movement that indicates its measure." This statement would be true if one stood at theoretic infinity (20 ft.) from the patient while making the examination, but as this is not feasible, and one must perforce sit within arm's reach, a metre at the outside, at least a +1.00 D. must be deducted, as also when the concave mirror is used.

The practice advocated of giving a weaker glass for reading in cases of myopia, is not generally followed in this country, the feeling being that it is better to encourage the ciliary muscle to do its physiologic work, therefore unless there is absolute loss of accommodation, most oculists prefer to give a full or nearly full correction for constant wear.

A price-list of lenses in London, presumably, shows that the United States does not need a tariff to protect her spectacle-makers from British competition as the prices quoted overage about 20 per cent. more than the best American opticians charge.

The chapter on color-blindness is concise and clear, and the essentials of the test have never been better explained in so few words.

The use of a cycloplegic is urged in most cases. This is gratifying to American ophthalmologists, as they have been the greatest advocates of their use. Recent literature seems to indicate that their British colleagues are now in agreement with them.

**Within The Mind Maze, or Mentonomy, the Law of the Mind,** by **Edgar Lucien Larkin,** Director of the Lowe Observatory. Published by the Standard Printing Co., Los Angeles, 1911. $1.00 (pages 336—523, continued from Radiant Energy, of the same series), cloth.

The sub-title, should obviously be changed to phrenonomy or psychonomy. This is an interesting book, of a speculative, metaphysical nature, leaning in places toward theology, based on the author's life work in astronomy and its fundamental sciences. Every man of a meditative nature, especially one at work along lines of natural science and mathematics, unless of the type that turns at once to details or is hopelessly prosaic and material, has evolved such thoughts in his leisure moments. Usually, for fear of being called a dreamer or from the false shame which most Christian races feel at displaying religious emotions except at stated times and places, he refrains from writing them down. Professor Larkin has held the mirror before us, where we can see our own naked mind. It is the fashion to decry speculation, to uphold the materialistic doctrine "Don't think, experiment." Having experimented, observed and studied material things, Professor Larkin boldly claims the right to think, to imagine, to apply mathematic concepts to the non-material.

---

**Text Book of Embryology** by **Frederick Randolph Bailey,** A.M., M.D., and **Adam Marion Miller,** A.M., of Columbia University; published by Wm. Wood & Co., second edition, 1911; 672 pages, 515 illustrations, cloth, $4.50.

The physician whose knowledge of embryology dates back a number of years will find a great mass of fact in this book with which he is unfamiliar and he will be impressed with the very different use of the loud and the soft pedal in different parts of the science. Formulae as to the size of the embryo at different periods of gestation have a distinct medico-legal or medico-social value in many cases and a clear understanding of embryology aids greatly in the conception of the development of neoplasms, in the limitations of various disease processes and in the spread and recurrence of disease. Both the authors and the publishers are to be congratulated on the general excellence of the work.

---

**Physicians' Visiting List,** 1912, P. Blakiston's Son & Co. Week-day dates for the year or perpetual, $1.25 to $1.50 according to number of patients. For 2 half year volumes, with week-day dates, $2.25—$2.50. Monthly edition, $1.00—$1.25.

This book is familiar to physicians and preserves the same convenient size and style in vogue for many years. We take this occasion, however late, to acknowledge the courtesy of the firm in presenting a copy to each member of the graduating class of the University of Pennsylvania, 23 years ago.

**Medical Chemistry and Toxicology,** by **James W. Holland,** M.D., Philadelphia; published by W. B. Saunders Co., Philadelphia, 3d edition, 1911. Octavo, 655 pages, 8 colored plates, 112 illustrations. Cloth, $3.00.

This work begins with a concise presentation of physics as a foundation for chemistry. It then deals with chemistry proper, and a little less than half of the book is devoted to organic and physiologic chemistry, treated in such a way as to make it readily available for diagnostic use. The colored diagram of the composition of a hydrocarbon flame is particularly instructive. The summary of color reactions of the urine, is of great value as an object lesson. That for gastric analysis makes us wonder whether our own color vision is normal. The work is replete with tables, diagrams, etc., of the utmost value in summing up scientific information and making it readily available.

---

**Progressive Medicine.** (Quarterly, paper covers), December 1, 1911. (Lea & Febiger, Philadelphia and New York, $6.00 yearly).

The present volume deals with the literature of the alimentary canal, etc., kidneys, genito-urinary diseases, certain phases of surgery and practical therapeutic advances. It is a most excellent review of periodic literature, not only for the typic "busy practitioner" but for the student who needs references for research. The only unfavorable criticism that can be made is that some of the editors make their personal judgement a little too prominent with a resulting Rooseveltian style. But, from experience, we realize that this fault is very difficult to avoid and it is not so great as the impartial chronicling of good, bad and indifferent opinions of the great mass of writers.

---

**Text Book of Pathology,** by **Francis Delafield,** M.D., LL.D., and **T. Mitchell Prudden,** M.D., LL.D., Columbia University, published by Wm. Wood & Co., New York, ninth edition 1911. 1114 pages. 13 plates and 687 illustrations, in black and color, cloth, $5.50.

As a matter of interest, we have looked up the third edition, which has been a useful companion for many years. The date of the ninth edition shows an increase of 22, the pages of over 500, the plates are all new and the illustrations are more than three times as numerous. Both authors have been increased by 600—LL.D. The price shows no increase at all. Seriously, it is difficult to review a book of this established reputation. It is not merely a pathology in the old, limited sense, but a bacteriology, a guide to post mortem examinations and, to a certain extent, a work on clinical pathologic diagnosis.

**A Manual of the Practice of Medicine,** 9th revised edition, by **A. A. Stevens,** A.M., M.D., Philadelphia; W. B. Saunders Co., Philadelphia, 1911; 12mo, 573 pages, illustrated, flexible leather, $2.50.

This is a very brief, systematic compend of practice, designed especially for the use of students, but by no means unworthy the attention of the graduate. The style is clear and the information imparted definite though, obviously, not sufficiently complete for an exhaustive study.

---

**Practice of Medicine, James M. Anders,** M.D., Ph.D., LL.D., Philadelphia; 10th edition, 1911, W. B. Saunders Co., Philadelphia and London. 1328 pages, illustrated, $5.50 cloth; $7.00 half morocco.

This well known and well liked text book has been revised to date. The usual arrangement is followed and it is well adapted both to the use of students and practitioners.

---

THE C. V. Mosby Company, of St. Louis, has announced the publication of a book on Pellagra, to be ready by January 1, 1912. This book is being prepared by Doctor Stewart R. Roberts, of Atlanta, Ga., who has just returned from Italy, where he studied the disease in its natural habitat. While in Europe the doctor made extensive research regarding the etiology and treatment of Pellagra, and the data contained in the book will reflect the latest and best work that has been done in connection with this disease, making it a reliable guide to those seeking information on the subject.

---

**International Clinics,** Vol. 3, 21st series, 1911, J. B. Lippincott Co., Philadelphia.

This publication, which has entered the gap between the magazine and the formal text book, maintains a high standard of excellence. A new department, Economics of Medicine, is included. The present volume contains an article by only one man in the special territory of the BUFFALO MEDICAL JOURNAL, Dr. A. L. Benedict, The Investigation of the Duodenum. Dr. Thomas R. Brown of Baltimore contributes an article on the relationship between Gastric and Urinary Acidity, closely following the lines of an article on the same general subject by the former, in Vol. 1 of the 18th series, 1908. This corroborates the novel fact that there is no practical "acid and alkaline tide" of urinary excretion, converse to acid and alkaline digestive secretion and that gastric and urinary acidity are not opposed in degree.

Note by the editor: (The allusion to our own work by the reviewer, is in accordance with the general rule to notice, in all exchanges and books for review, the work of men in western New York, corresponding approximately to the 5th to the 8th District Branches. As the article in question consists largely of confessions of ignorance and of failures to obtain the brilliant results claimed by some, we feel that it is not immodest to let the review stand.)

---

**The Way with the Nerves,** Letters to a Neurologist on Various Modern Nervous Ailments, Real and Fancied, with Replies thereto Telling of their Nature and Treatment, by **Dr. Joseph Collins** of New York. Published by C. P. Putnam's Sons, New York & London, 1911. 313 pages, $1.50.

It is difficult to do justice to this book. It is not adapted to lay use except in the cases in which the physician wishes his patient to understand his own neurotic condition and is willing also for him to realize some of the foibles of the profession entertainingly discussed under the heading of the Bedside Manner. Nor will the neurologist nor even the shrewd general practitioner find much that is absolutely new to him. Nevertheless, it contains a great deal of valuable information and many hints as to the tactful management of neurotic cases.

---

Fifteen months ago Mrs. X. came to me for treatment, giving the following history: Six years previously she had a miscarriage, since which she has been troubled with a profuse leuckorrhea of a very foul odor. At her menstrual period she suffered greatly and flowed excessively. On examination the cervix was found to be nearly four times its normal size and so badly eroded as to have every appearance of a cancer and had been mistaken for such by one physician. The uterus was soft and boggy and very much enlarged. She had been to the hospital on two occasions and each time had been curetted, but this seemed only to aggravate the general condition. For over a year I treated her with every means at hand but to no purpose. I was making preparation for an operation, which would have meant the removel of the uterus, when my attention was drawn to Glyco-Thymoline and I determined to give it a thorough trial before operative measures were to be further introduced. An intrauterine douche of Glyco-Thymoline in a 25% hot solution was administered and lamb's wool tampons saturated with pure Glyco-Thymoline were used. She began to improve from the first application. The leuckorrhea became less and the odor disappeared entirely. The cervix took on a healthy look. The uterus decreased in size and became firm; in fact she is now nearly well after nine weeks' treatment with Glyco-Thymoline.

